# The Roles of Epigenetics Regulation in Bone Metabolism and Osteoporosis

**DOI:** 10.3389/fcell.2020.619301

**Published:** 2021-01-25

**Authors:** Fei Xu, Wenhui Li, Xiao Yang, Lixin Na, Linjun Chen, Guobin Liu

**Affiliations:** ^1^College of Medical Technology, Shanghai University of Medicine and Health Sciences, Shanghai, China; ^2^Collaborative Innovation Center, Shanghai University of Medicine and Health Sciences, Shanghai, China; ^3^College of Clinical Medicine, Shanghai University of Medicine and Health Sciences, Shanghai, China; ^4^Traditional Chinese Vascular Surgery, Shuguang Hospital Affiliated to Shanghai University of Traditional Chinese Medicine, Shanghai, China; ^5^College of Public Health, Shanghai University of Medicine and Health Sciences, Shanghai, China

**Keywords:** epigenetics, osteoporosis, DNA methylation, histone modification, non-coding RNA

## Abstract

Osteoporosis is a metabolic disease characterized by decreased bone mineral density and the destruction of bone microstructure, which can lead to increased bone fragility and risk of fracture. In recent years, with the deepening of the research on the pathological mechanism of osteoporosis, the research on epigenetics has made significant progress. Epigenetics refers to changes in gene expression levels that are not caused by changes in gene sequences, mainly including DNA methylation, histone modification, and non-coding RNAs (lncRNA, microRNA, and circRNA). Epigenetics play mainly a post-transcriptional regulatory role and have important functions in the biological signal regulatory network. Studies have shown that epigenetic mechanisms are closely related to osteogenic differentiation, osteogenesis, bone remodeling and other bone metabolism-related processes. Abnormal epigenetic regulation can lead to a series of bone metabolism-related diseases, such as osteoporosis. Considering the important role of epigenetic mechanisms in the regulation of bone metabolism, we mainly review the research progress on epigenetic mechanisms (DNA methylation, histone modification, and non-coding RNAs) in the osteogenic differentiation and the pathogenesis of osteoporosis to provide a new direction for the treatment of bone metabolism-related diseases.

## Highlights

- We summarize the research progress of epigenetic mechanisms in bone metabolism and osteoporosis.
- We summarize the role of DNA methylation in the osteogenic differentiation and osteoporosis.
- We summarize the role of histone modification in the osteogenic differentiation and osteoporosis, including histone methylation and histone acetylation.
- We summarize the role of non-coding RNA in the osteogenic differentiation and osteoporosis, including lncRNAs, miRNAs, and circRNAs.

## Introduction

The integrity of human bones is maintained by the repeated, spatiotemporal coupling of bone resorption and bone formation, which is called bone remodeling (Kenkre and Bassett, 2018; Intemann et al., 2020). When the balance between bone formation and bone resorption is disturbed and the ratio of bone resorption to bone formation is increased, the resulting progressive bone loss can lead to a degenerative bone metabolic disease, that is termed osteoporosis (OP), which is characterized by decreased bone mineral density (BMD), degeneration of bone microstructure, and increased bone fragility and fracture risks (Morris et al., 2012; Siris et al., 2014; Kenkre and Bassett, 2018). Genomics are an important factor in determining the risk of BMD and OP, and numerous polymorphisms of genes related to bone metabolism are associated with bone mass, OP susceptibility and fracture risk. However, these variations in known gene sequences add up to only explain part of the pathogenesis of osteoporosis (Hsu and Kiel, 2012; Bjornerem et al., 2015). With global social economy developments and improved living standards, the prevalence of OP continually increases. According to relevant statistics, there are more than 10,000 patients with OP in China, and a certain percentage of the population suffers from OP of different degrees. The pathogenesis of OP is related to a variety of factors, including age, sex, endocrine hormone levels, living habits, dietary factors, and heredity (Foger-Samwald et al., 2020; Gkastaris et al., 2020).

**FIGURE 1:**
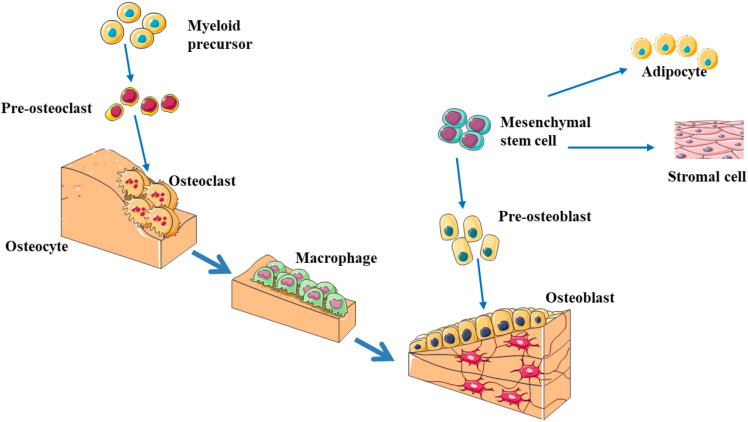
The schematic diagram of bone remodeling process (Chen et al., 2018; Foger-Samwald et al., 2020; Gkastaris et al., 2020; Zou et al., 2020). Bone remodeling process is initiated by osteoclasts that solubilize bone mineral and degrade the matrix (resorption phase). Osteoclasts originate from hematopoietic stem cells which differentiate first into pre-osteoclast cells which fuse to form multinucleated cells (activated osteoclasts). Monocytes/macrophages remove debris (reversal phase), followed by a bone formation phase performed by osteoblasts, producing osteoid matrix which will mineralize. Growth factors are released from the bone matrix during resorption, which increase the pre-osteoblast population in order to replace damaged bone surfaces.

Bone formation and bone resorption are the two basic processes that maintain normal bone reconstruction, and osteoblasts and osteoclasts play an important role in this process, where osteoblasts promote bone formation and osteoclasts promote bone absorption (Figure 1; Chen et al., 2018; Foger-Samwald et al., 2020). The precise regulation and balance of osteoblasts and osteoclasts in terms of function and quantity help maintain the normal bone reconstruction process, and abnormal differentiation of osteoblasts and osteoclasts leads to the imbalance of bone remodeling (Gkastaris et al., 2020; Zou et al., 2020). The resulting decrease in bone formation and/or increase in bone resorption can lead to a decrease in bone mass, which may lead to OP. With the growth of the aging population, rapid socioeconomical development and lifestyle changes, the incidence of OP is also increasing. Brittle fractures caused by OP have become an important public health problem because of the associated high morbidity, mortality and disability rates and consumption of a large amount of social public health resources (Letarouilly et al., 2019; Williams and Sapra, 2020; Yang et al., 2020).

Epigenetics generally refers to heritable phenotypic changes that do not involve alterations in the DNA sequences. Although the genotype does not change, the phenotype undergoes hereditary changes, including DNA methylation, histone modification, and non-coding RNA (ncRNAs) alterations (Yang and Duan, 2016; Letarouilly et al., 2019; Li et al., 2020). Epigenetics play important regulatory roles in many biological processes, such as tissue-specific gene expression, chromosome inactivation, genomic imprinting and cell differentiation (Yang and Duan, 2016). An increasing number of studies have shown that epigenetic abnormalities are important causes of malignant tumors, metabolic diseases, somatic diseases and autoimmune diseases (Letarouilly et al., 2019; Pang et al., 2020; Yang et al., 2020; Zovkic, 2020). In recent years, studies have shown that epigenetics are involved in the regulation of bone formation and can significantly affect the differentiation of osteoblasts and osteoclasts (Letarouilly et al., 2019; Li et al., 2020). The application of epigenetics to the study of mechanisms related to bone biology and bone metabolism and to the exploration of mechanisms regulating the differentiation and proliferation of osteoblasts and osteoclasts is of great significance for understanding the etiology and pathogenesis of metabolic bone diseases, such as OP, as well as for developing appropriate prevention and treatment strategies of these diseases. In this article, we will review the research progress of epigenetic mechanisms in bone metabolism and OP.

## DNA Methylation and OP

**FIGURE 2:**
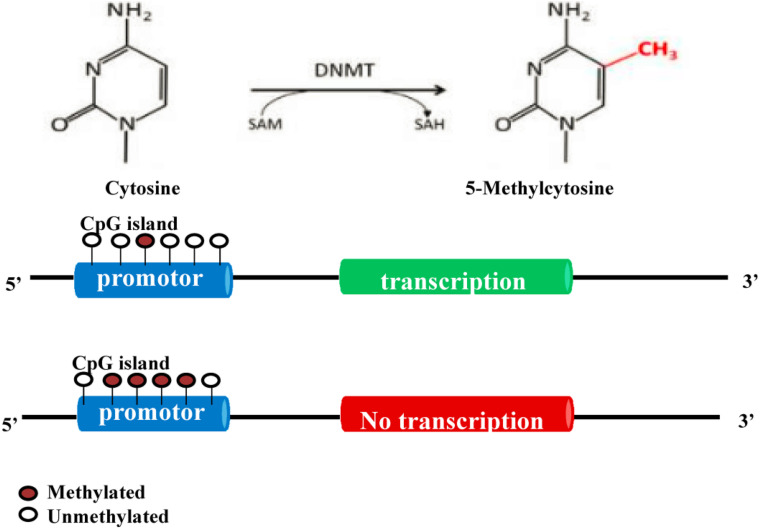
Molecular mechanism of DNA methylation (Yang and Duan, 2016; Letarouilly et al., 2019; Ahmed et al., 2020; Wang et al., 2020; Xu et al., 2020). DNA methylation modification is mainly controlled by DNMT proteins. S-adenosylmethionine is used as the methyl donor to methylate the cytosine on CpG islands. Normally, the CpG island of a gene is in an unmethylated state. Methylation of the cytosines in the CpG island can inhibit the expression of this gene.

**FIGURE 3:**
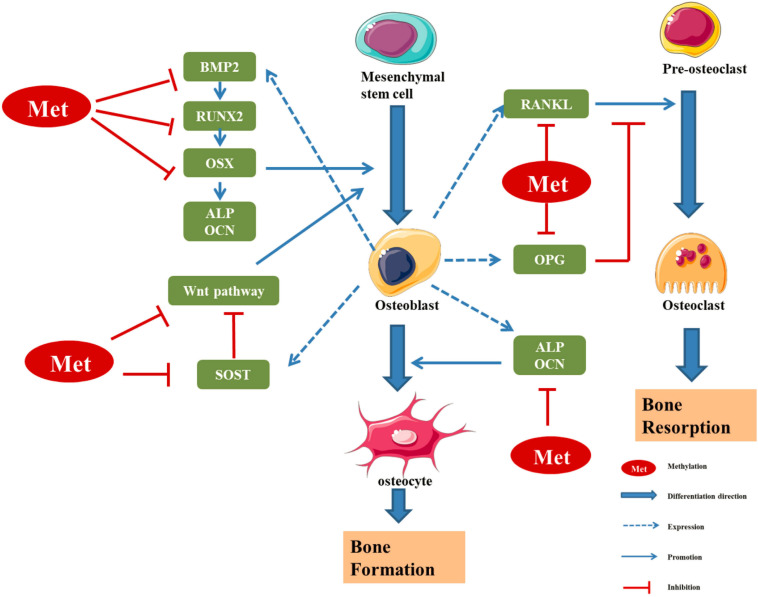
Regulatory effect of methylation levels of bone metabolism-related genes on bone formation (Kitazawa and Kitazawa, 2007; Delgado-Calle et al., 2012; Fu et al., 2013; Cao et al., 2019; Kalkan and Becer, 2019; Licini et al., 2019; Raje and Ashma, 2019; Chen D. et al., 2020; Kim et al., 2020). RUNX2 and OSX are specific transcription factors, which synergistically regulate the expression of bone-specific genes, including those encoding ALP, type I collagen and OCN. BMP2 is a key bone growth factor that can stimulate MSCs to differentiate into osteoblasts by inducing the expression of genes such as those encoding RUNX2, OSX, and OCN. The hypermethylation of the BMP2 promoter region in osteoblasts leads to downregulation of bone formation markers. SOST, a glycoprotein mainly secreted by osteoblasts, can inhibit osteoblast differentiation by inhibiting Wnt signal transduction and negatively regulates bone formation. The hypermethylation of SOST can inhibit SOST gene translation and promote the osteoclast differentiation. DNA methylation can regulate the transcription and expression of Wnt/β-catenin signalling pathway molecules, so as to regulate the differentiation and function of osteoblasts. OPG/RANK/RANKL is the main regulator of the balance between osteoblasts and osteoclasts. The methylation of these gene promoter regions can regulate the expression of corresponding genes, thus affecting the differentiation and function of osteoblast/osteoclast, and finally affecting the dynamic balance between bone formation and bone absorption in the process of bone remodeling (Niu et al., 2020).

DNA methyltransferases (DNMTs) play an important role in the processes of embryogenesis, development and methylation. It plays a considerable role in genome stability, gene expression and individual development in both prokaryotes and eukaryotes (Yang and Duan, 2016; Letarouilly et al., 2019). Modifications of DNA methylation are mainly controlled by DNMT family proteins, and S-adenosylmethionine is used as the methyl donor for cytosine residues on CpG islands (Xu et al., 2020). Normally, CpG islands on genes are present in an unmethylated state, and methylation of the cytosines on these islands can inhibit the expression of the gene (Niu et al., 2020). A certain hypomethylation status is conducive to the expression of related genes, while a hypermethylation status can lead to gene silencing (Figure 2; Xu et al., 2020). Increasing studies have shown that DNA methylation can regulate the differentiation and apoptosis of osteoblasts and osteoclasts to play an important role in the pathomechanism of OP (Table 1 and Figure 3; Letarouilly et al., 2019).

**TABLE 1:** Osteogenic differentiation markers regulated by DNA methylation modification.

Genes	Methylation level during osteogenic differentiation	Gene function in osteogenesis
RUNX2	Low	TF, promote the expression of target genes and osteogenic differentiation (Zhang R. P. et al., 2011; Wakitani et al., 2017)
OSX	Low	TF, promote the expression of target genes and osteogenic differentiation (Zhang R. P. et al., 2011; Farshdousti Hagh et al., 2015)
BMP2	Low	Bone growth factor, promote osteogenic differentiation (Fu et al., 2013; Raje and Ashma, 2019)
SOST	High	Glycoprotein, inhibit osteogenic differentiation (Reppe et al., 2015a; Cao et al., 2019)
ALP	Low	Hydrolyze phosphate ester to provide necessary phosphoric acid for the deposition of hydroxyapatite, and at the same time hydrolyze pyrophosphate to remove its inhibitory effect on bone salt formation (Delgado-Calle et al., 2011; Yang and Duan, 2016; Licini et al., 2019)
OCN	Low	Maintain normal bone mineralization (Villagra et al., 2002; Licini et al., 2019)
Frizzled1	Low	Activate the wnt pathway and promote osteogenic differentiation (Wu et al., 2019)
RANKL	High	Stimulate osteoclast differentiation and promote bone resorption (Ghayor and Weber, 2016; Behera et al., 2018)
OPG	Low	Inhibit osteoclast differentiation (Ghayor and Weber, 2016; Wang et al., 2018)
LOX	Low	Promote osteogenic differentiation (Thaler et al., 2011)
ESR1	Low	Promote osteogenic differentiation (Penolazzi et al., 2004)
DLX5	Low	Promote osteogenic differentiation (Lee J. Y. et al., 2006; Li et al., 2009)
Alu elements	High	Negatively correlated with bone formation (Jintaridth et al., 2013)

*TF, transcription factor; RUNX2, Runt-related transcription factor 2; OSX, osterix; BMP2, bone morphogenetic protein 2; SOST, sclerostin; ALP, alkaline phosphatase; OCN, osteocalcin; OPG, Osteprotegerin; RANKL, nuclear factor-κB ligand; LOX, lysyl oxidase; ESR1α, estrogen receptor alpha; DLX5, distal-less homeobox 5.*

### Osteogenic Differentiation Markers Regulated by DNA Methylation Modification

#### DNMTs

Four known DNMT subtypes (DNMT1, DNMT3a, DNMT3b, and DNMT3L) exist in mammalian cells, the first three of which are active DNMTs (Ahmed et al., 2020; Wang et al., 2020). The major DNA methylation inhibitors can be divided into two broad classes: nucleoside analogies, such as 5-aza-2’-deoxycytidine (5-Aza-dC) and 5-Aza-C, and non-nucleoside analogs, including procaine, homocysteine (Hcys). Among them, 5-Aza-dC and 5-Aza-C are the most widely used DNA methylation inhibitors. Studies have shown that DNMTs plays an important role in bone biology, and methylation inhibitors can interfere with the osteogenesis process.

Zhou et al. (2009) interfered with the osteogenic differentiation of mesenchymal stem cells (MSCs) with 5-AzaC, and found that 5-AzaC demethylated the genome, increased the expression of osteogenic-related genes and effectively promoted the osteogenic differentiation. It has also been reported that 5-Aza-dC can demethylate distal-less homeobox 5 (DLX5) and osterix (OSX) gene promoters and upregulate the expression of osteogenic markers, such as alkaline phosphatase (ALP) and osteocalcin (OCN) (Farshdousti Hagh et al., 2015). Procaine has no cytotoxic effect, and has been used in studies on vascular smooth muscle cell (VSMC) calcification. Procaine was also shown to reduce the methylation level of the smooth muscle protein 2α (SM2α) promoters, increase SM2α expression, inhibit DNMT activity, and block vascular calcification (Montes de Oca et al., 2010). Another methylation inhibitor, Hcys, promotes osteoblast differentiation (Turecek et al., 2008; Thaler et al., 2010). After Hcys intervention, the expression of lysyl oxidase (LOX) gene promoter CpG island was significantly increased, which inhibited LOX expression, interfered with the formation of bone matrix, and ultimately affected the differentiation of osteoblasts (Thaler et al., 2011). Nishikawa et al. (2015) found that DNMT3a could promote osteoclast differentiation and bone absorption by inhibiting interferon regulatory factor 8 (IRF8), which is negatively regulates osteoclast differentiation. DNMT3a inhibits IRF8 mainly by increasing the methylation of the remote regulatory element IRF8, and increasing the concentration of S-adenosine methionine can promote its methylation. Specific deletion of DNMT3a in osteoclasts or treatment with the DNMT3 inhibitor TF-3 protected mice against ovariectomy-induced bone loss. Liu H. et al. further found that the osteolytic changes in myeloma patients were related to the upregulation of IRF8 methylation by the thymidine phosphorylase (TP) secreted by myeloma cells, and the expression of IRF8 was decreased, which further enhanced bone resorption, suggesting that epigenetics could be a potential target for the treatment of bone disease (Liu H. et al., 2016).

#### RUNX2 and OSX

Runt-related transcription factor 2 (RUNX2) and OSX are specific transcription factors necessary for bone formation and osteoblast differentiation, in which OSX is the downstream target of RUNX2 (Komori, 2019; Chen D. et al., 2020). During the osteoblast differentiation of MSCs, the level of RUNX2 methylation was decreased, suggesting that RUNX2 methylation plays an important regulatory role in osteoblast differentiation (Wakitani et al., 2017). However, Farshdousti Hagh et al. (2015) found that in the process of osteogenic differentiation, the methylation states of the promoter regions of RUNX2 and DLX5 did not change, while the methylation level in the promoter region of OSX changed dynamically, suggesting that the epigenetic regulation of OSX may play a major role in the osteogenic differentiation of MSCs. Similarly, in the osteogenic induction of adipose tissue-derived MSCs, the expression levels of the osteogenic-specific genes Runx2 and OSX were upregulated. DNA methylation sequencing further confirmed that the promoter regions of Runx2 and OSX exhibited significantly decreased DNA methylation levels, and the DNA methylation levels were significantly correlated with gene expression (Zhang R. P. et al., 2011). Inhibition of DNA demethylase reversed the expression levels of these genes, suggesting that Runx2 and OSX are primarily regulated by DNA methylation mechanisms in the osteogenic differentiation of adipose tissue-derived MSCs (Zhang R. P. et al., 2011).

#### BMP2

Bone morphogenetic protein 2 (BMP2) is a key bone growth factor that can stimulate MSCs to differentiate into osteoblasts (Chen et al., 2012; Kamiya, 2012). Studies shown that hypermethylation of the BMP2 promoter in osteoblasts could inhibit the expression of bone formation-related genes (Fu et al., 2013; Raje and Ashma, 2019). Researchers demonstrated that the level of methylation 267 bp upstream of the BMP2 transcription start site in patients with OP was significantly correlated with the degree of OP, which led to downregulated transcriptional activity and gene expression of the BMP2 promoter (Raje and Ashma, 2019).

#### ALP and OCN

Alkaline phosphatase and osteocalcin are secreted mainly by osteoblasts, and both are used as the most common bone formation markers to assess osteogenic activity (Licini et al., 2019). It was found that the ALP promoter regions in human osteoblasts and osteoblasts had opposite DNA methylation profiles, as that in osteoblasts was hypomethylated, while that in osteoblasts was hypermethylated, indicating that the DNA methylation pathway could inhibit the expression of ALP during the process of osteogenic differentiation (Delgado-Calle et al., 2011; Yang and Duan, 2016). OCN is an important marker of osteogenic differentiation (Licini et al., 2019). During the differentiation of primary osteoblast, the methylation of OCN promoter is gradually decreased, suggesting that the hypomethylation of OCN can promote osteogenic differentiation (Villagra et al., 2002).

#### Alu Elements

Alu elements are short interspersed elements (SINEs) which are unique to primates. They play a special role in human genome reorganization, variable splicing and post-mRNA transcription regulation (Kim et al., 2016; Jiang et al., 2019). During the period of rapid growth in children, the level of Alu element methylation is significantly increased (Rerkasem et al., 2015). Jintaridth et al. (2013) showed that the hypomethylation of Alu elements is correlated with the occurrence of osteoporosis in postmenopausal women, which suggested the relationship between the hypomethylation of the whole genome and aging-related diseases. These studies reflected that the methylation level of the Alu elements is negatively correlated with bone formation and the hypomethylation level of Alu elements may be related to the occurrence of OP.

#### The Wnt/β-Catenin Signaling Pathway

The key downstream effector protein in the Wnt/β-catenin signaling pathway is the transcriptional activator β-catenin. In the absence of Wnt stimulation, the cytosolic β-catenin level keeps low through phosphorylation by the APC (adenomatous polyposis coli)–Axin–GSK-3β (glycogen synthase kinase 3β) destruction complex and ubiquitin-dependent degradation in the proteasome. Upon Wnt stimulation, the destruction complex is destabilized, which leads to accumulation and nuclear translocation of the cytosolic β-catenin to activate the transcription of Wnt/β-catenin-responsive target genes (Hartmann, 2006; Cao et al., 2018). The genes in the Wnt signaling pathway are also regulated by DNA methylation. During the differentiation of BMSCs into osteoblasts, the level of methylation in receptor tyrosine kinase-like orphan receptor 2 (ROR2) promoter region of the Wnt signaling pathway was shown to be reduced (Tarfiei et al., 2011). In patients with diffuse idiotic bone hypertrophy, the osteogenic characteristics of MSCs isolated from the spinal cord ligament were promoted by unmethylated Wnt5a (Chiba et al., 2015). These studies showed that DNA methylation regulates the expression and activity of molecules in the Wnt/β-catenin signaling pathway, thus participating in the pathological mechanisms of OP.

#### The OPG/RANKL/RANK Signaling Pathway

Bone remodeling is closely regulated by the RANKL-RANK-OPG system, and the current studies on the relationship between DNA methylation and osteoporosis mostly focus on this. Osteprotegerin (OPG) and nuclear factor-κB (NF-κB/RANK) ligand (RANKL) are important determinants of bone quality and strength. RANKL binds to RANK, a receptor present in osteoclast lines, which activates osteoclast formation, activation, and survival. The binding of RANKL to OPG can prevent excessive bone resorption and avoid the interaction between RANKL and RANK. OPG/RANKL/RANK is a signaling channel that can regulate the differentiation of osteoclasts and is one of the most important signaling pathway for bone metabolism pathways (Chen et al., 2018). DNA methylation of RANKL and its soluble receptor OPG plays an important role in the regulation of osteoclast differentiation. Quantitative methylation of all types of bone cells and pyrolytic acid sequencing analysis showed that methylation of the transcription initiation regions of RANKL and OPG inhibited the transcription of RANKL and OPG genes (Kitazawa and Kitazawa, 2007; Delgado-Calle et al., 2012; Kalkan and Becer, 2019). Therefore, the methylation regulation of OPG/RANK/RANKL plays an important role in osteogenic differentiation (Ghayor and Weber, 2016).

### Whole Genome DNA Methylation in Osteoporosis

The emergence of next-generation sequencing technology provides an unprecedented opportunity to analyze DNA methylation patterns at the whole genome level (Niu et al., 2020). Delgado-Calle et al. (2013) used Illumina 27k methylation chip to determine genome-wide methylation profiles of bone from patients with osteoporotic hip fractures. The results revealed 241 CpG sites, located in 228 genes, with significant differences in methylation. These regions were enriched in genes associated with cell differentiation and skeletal embryogenesis (Delgado-Calle et al., 2013). de la Rica et al. (2013) compared the DNA methylation profiles of monocytes (MOs) and derived osteoclasts (OCs) following M-CSF and RANKL stimulation. They found that osteoclastogenesis was associated with the drastic reshaping of the DNA methylation landscape. Hypermethylation and hypomethylation occur in many relevant functional categories and key genes, including those whose functions are crucial to OC biology, which strongly proves the key role of DNA methylation regulation mechanism in osteoclast differentiation (de la Rica et al., 2013). In 2016, Alvaro del Real et al., used the Infinium 450K bead array and RNA sequencing to determine DNA methylation research and transcriptome analysis of human mesenchymal stem cells (hMSCs) isolated from the femoral heads of patients with osteoporotic fractures or osteoarthritis. The results showed that the epigenome-wide signature of hMSCs from fracture patients shows differentially methylated regions in comparison with hMSCs derived from OA patients. These regions are associated with several genes involved in MSC proliferation and differentiation, such as RUNX2/OSX (Del Real et al., 2017). Reppe et al. combined transcript profiling with DNA methylation analyses in bone. RNA and DNA were isolated from 84 bone biopsies of postmenopausal donors varying markedly in bone mineral density (BMD). Among the top 100 genes most significantly associated with BMD, four transcripts representing inhibitors of bone metabolism—MEPE, SOST, WIF1, and DKK1—showed correlation to a high number of methylated CpGs (Reppe et al., 2015b, 2017).

In 2017, Fernandez-Rebollo et al., analyzed genome wide DNA methylation profiles of peripheral blood from patients with manifest primary osteoporosis and non-osteoporotic controls. Statistical analysis did not reveal any individual CpG sites with significant aberrant DNA methylation in osteoporosis. Therefore, the author indicated that osteoporosis is not reflected by characteristic DNA methylation patterns of peripheral blood, which could not be used as a biomarker for osteoporosis (Fernandez-Rebollo et al., 2018). Morris et al. (2017) performed a large-scale epigenome-wide association study of BMD using the Infinium HumanMethylation450 array to measure site-specific DNA methylation in up to 5515 European-descent individuals. They identified one CpG site, cg23196985, significantly associated with femoral neck BMD. But, this association has not been repeated in another population, suggesting future epigenomic studies of musculoskeletal traits measure DNA methylation in a different tissue with extended genome coverage (Morris et al., 2017). On the contrary, Cheishvili et al. (2018) used Illumina Infinium human methylation 450K analysis to delineate the DNA methylation signatures in whole blood samples of 22 normal women and 22 postmenopausal osteoporotic women (51 to 89 years old) from the Canadian Multicenter Osteoporosis Study (CaMos) cohort. Analysis of the female participants with early and advanced osteoporosis resulted in the generation of a list of 1233 differentially methylated CpG sites when compared with age-matched normal women. Heat map and hierarchical clustering analysis showed that the most significant differentially methylated 77 CpG sites are associated with OP and can be detected even in early stages of OP in white blood cells. As DNA methylation patterns are highly tissue specific, whether peripheral blood DNA methylation pattern can be used as OP biomarkers is still controversial.

The current direction of research on DNA methylation and OP is to elucidate the pattern of genomic methylation in osteoblasts, target genes, and the relationship between methylation and bone density changes. These studies will identify new biological markers for bone mineral density changes and OP risk factors in humans and yield groundbreaking results in the field of OP. In addition to these prospective studies on methylation in the field of bone metabolism, the mechanism of methylation in human osteoblasts at the genome-wide level is still poorly understood, and further studies are needed to provide a new targeted therapy for OP.

## Histone Modification and OP

Histones, for which five types exist (H1, H2A, H2B, H3, and H4) are small-molecule proteins that are rich in positively charged basic amino acids (arginine and lysine) and can interact with negatively charged phosphate groups in DNA. Histone chemical modification occurs at the N-terminal tail of the protein, especially for H3 and H4, promoting changes in chromatin structure. The histone tail is composed of 20 amino acids and extends from the nucleosome at the turning point of DNA. The nucleosome is a complex of several histone subunits and DNA that protects DNA and epigenetic information. The post-translational modification of histones is a key step in epigenetic regulation, as it affects lineage submission and gene expression. Refolding covalent histone modifications occur most often at the amino and carboxyl ends of chemically unstable amino acid residues (e.g., lysine, arginine, serine, threonine, tyrosine, and histidine) as well as during histone inversion or in the globular domains of nucleosomal nuclei (Duman et al., 2020; Ren et al., 2020). Each modified histone residue carries specific information, and in general, H3K3 exists in a stable transcriptional state. Transmission of information can recruit binding factors that influence histone modification and remodeling through a variety of mechanisms, including changing the interaction among histones themselves or between histones and DNA (Longbotham et al., 2020). Below, research on histone acetylation and methylation in the context of osteogenic differentiation and OP is reviewed.

### Histone Acetylation and OP

Studies have shown that histone modifications of euchromatin are characterized by high levels of acetylation and trimethylated H3K4, H3K36, and H4K20 (Longbotham et al., 2020). Heterochromatin shows low acetylation and high methylation of H3K9, H3K27, and H4K20. Most histone modifications are regulated by modifying enzymes that can promote and reverse these specific modifications. These include histone acetyltransferases (HATs) and histone deacetylases (HDACs) (Xu et al., 2020). According to their structural and functional characteristics, HDACs can be divided into four categories: class I includes HDAC1, 2, 3, and 8; class II includes HDAC4-7, 9, and 10; class III includes sirt1-7; and class IV includes HDAC11. In addition, many transcription factors can affect the activities of HATs and HDACs, thus affecting the balance between acetylation and deacetylation and ultimately affecting the expression of target genes (Ren et al., 2020).

Different HDAC antagonists have been used to investigate the relationships of high acetylation of total histones with both osteoblast differentiation and gene expression. These HDAC antagonists include trichostatin (TSA), suberoylanilide hydroxamic acid (SAHA), entenol (MS-275), sodium butyrate and valproic acid. *In vitro* experiments showed that blocking class I and class II HDACs at the same time or blocking class I HDACs alone could promote osteoblast maturation, bone mineralization and the expression of genes related to osteoblast differentiation and maturation, such as type I collagen, osteopontin (OPN), OCN, ALP, OSX, and RUNX2 (Schroeder and Westendorf, 2005; Schroeder et al., 2007; Schröder et al., 2020). Interestingly, TSA-mediated acetylation of histones H4 and H3 in the RANKL promoter region resulted in increased expression of RANKL (Fan et al., 2004). Animal models of bone loss showed bone mass recovery under the action of MS-275, while healthy animal models showed bone loss under the action of SAHA or valproic acid (Senn et al., 2010; Kim H. N. et al., 2011; McGee-Lawrence et al., 2011). Furthermore, the effect of valproic acid on bone tissue was further explored in patients with mental disorders who used valproic acid, revealing that BMDs were bone mineral density decreased and fracture risks were increased in these patients (Bradley et al., 2015; Shreya et al., 2019).

Osteocalcin is a bone tissue-specific protein that can bind to calcium, and its level of expression in plasma can be used as a marker of bone formation. Furthermore, its expression can determine the differentiation and activity of osteoblasts. When OCN transcription is active, histones H3 and H4 of the OCN promoter are acetylated, while histones H3 and H4 are acetylated at low levels when OCN transcription is inactive (Shen et al., 2002; Seuter et al., 2013; Pickholtz et al., 2014). HDAC3 can inhibit the activation of the OCN promoter by interacting with RUNX2, resulting in a decrease in OCN transcription activity (Sierra et al., 2003; Choo et al., 2009). Lamour et al., demonstrated that HDAC3 can reduce the acetylation level of the bone sialoprotein promoter H3, thus decreasing its expression and confirming the inhibitory effect of HDAC3 (Choo et al., 2009). TGF-β, as a negative regulator of bone formation, can interact with RUNX2 by recruiting HDAC4 and HDAC5, leading to histone H4 acetylation in the OCN promoter region (Kang et al., 2005). HDAC4 and HDAC5 can also directly reduce the acetylation level of RUNX2, thus decreasing its protein stability and transcriptional activity. In addition to HDAC3, HDAC4, and HDAC5, HDAC1 is also considered to be a regulator of osteoblast differentiation. Lee H. W. et al. found that the H3 and H4 hyperacetylation of the OSX and OCN promoters was due to a decrease in HDAC1 recruitment and an increase in p300 binding (Lee H. W. et al., 2006). Based on the above conclusions, HDAC activity plays an important regulatory role in osteogenic differentiation (Table 2).

**TABLE 2:** Histone deacetylases, target histones and their roles in the osteoblast differentiation.

HDACs	Target histones	Function
HDAC1	H2A, H2B, H3, H4	Regulate transcription and osteoblast differentiation (Rahman et al., 2003; Lee H. W. et al., 2006)
HDAC2	H2A, H2B, H3, H4	Regulate osteoblast differentiation (Rahman et al., 2003)
HDAC3	H2A, H2B, H3K27, H3, H4	Inhibit osteoblasts gene expression (Hesse et al., 2010)
HDAC4	H2A, H2B, H3K9, H3, H4	Regulate transcription, hypertrophy and ossification of chondrocytes (Kang et al., 2005; Jeon et al., 2006)
HDAC5	H2A, H2B, H3K9, H3, H4	Inhibit osteoblasts gene expression (Jeon et al., 2006; Kim J. H. et al., 2011)
HDAC6	H2A, H2B, H3K9, H3, H4	Regulate Runx2 activity and gene expression (Kim J. H. et al., 2011)
HDAC7	H2A, H2B, H3K9, H3, H4	Inhibits osteoblasts gene expression (Pham et al., 2011)
HDAC8	H2A, H2B, H3K9, H3, H4	Maxillofacial bone development (Fu et al., 2014)
SIRT1	H3, H4	Regulate proliferation of BMMSCs and osteoblastdifferentiation (Shakibaei et al., 2011; Edwards et al., 2013)
SIRT6	H3, H4, H3K9, H3K56	Regulate chondrocyte proliferation (Piao et al., 2013; Nagai et al., 2015)

#### Sirtuin 1 (SIRT1)—an Important Regulator of Bone Metabolism

**FIGURE 4:**
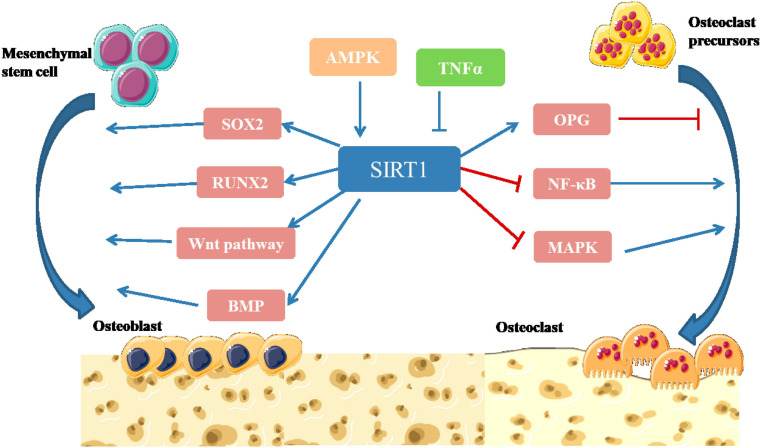
Illustration of SIRT1 signaling pathway in bone remodeling (Cohen-Kfir et al., 2011; Shakibaei et al., 2011; Edwards et al., 2013; Feng et al., 2014; Iyer et al., 2014; Kim et al., 2015; Liu J. et al., 2016). SIRT1 participates in intracellular energy metabolism through AMPK signaling pathway, and regulates bone metabolism in an AMPK-dependent manner. SIRT1 can promote the differentiation of MSCs into osteoblasts by directly deacetylating SOX2. SIRT1 can deacetylate β-catenin and promote β-catenin to accumulate in the nucleus, thus further activating Wnt pathway to promote Osteogenesis differentiation. SIRT1 can activate the transcription factor RUNX2 and promote the differentiation of MSCs into osteoblasts. Activation of SIRT1 can significantly increase the expression level of BMP-2 and BMP-7 to promote bone repair. SIRT1 inhibits NF-κB degradation, downregulates NF-κB signaling and inhibits bone resorption. Activation of SIRT1 can significantly inhibit the activity of MAPK signaling pathway by inhibiting the expression of prostaglandin, promote the expression of OPG and inhibit osteoclast generation and bone resorption (Yang and Tang, 2019).

Sirtuin 1 is highly homologous to the silence and yeast information adjustment factor 2 (Sir2) protein, which belongs to class III HDAC1 (Yang and Tang, 2019). SIRT1 gene is located on chromosome 10, and contains 8 introns and 9 exons, which encode the 500-amino acid Sirtuin 1 protein. The structure of SIRT1 is relatively conservative (Yang and Tang, 2019). The C-terminal domain consists of 25 amino acid residues, which constitute the core region of Sirtuin 1, namely, the deacetylation functional area. Sirtuin 1 is widely distributed and is mainly localized in the nucleus but also travels to the cytoplasm. SIRT1 targets many post-transcriptional regulators, including p53, forkhead box O (FoxOs), NF-κB, and peroxidase proliferator activator receptor (PPAR), which are associated with numerous human diseases (Feng et al., 2014; Kim et al., 2015; Liu J. et al., 2016). Studies have shown that SIRT1 can promote the differentiation of osteoblasts, inhibit the formation of osteoclasts, regulate bone reconstruction, and affect bone metabolism (Figure 4) (Liu J. et al., 2016).

In the physiological state, bone formation and bone resorption alternate to achieve balance, and osteoblasts and osteoclasts play roles in this process; during the aging process, the incidence of OP increases because bone absorption occurs more readily than bone formation. The expression of Sirtuin 1 is closely related to osteogenic factors. After bilateral ovariectomized rats were treated with resveratrol, the serum ALP and OCN levels were increased, and the BMD was increased. Resveratrol could promote osteogenic differentiation through the SIRT1/NF-κB pathway (Feng et al., 2014). SIRT1 can bind FOXO3a to form a complex and increase FOXO3a-dependent transcriptional regulation function. FOXO3a contains the binding domain of the RUNX2 promoter and can promote the expression of RUNX2 (Gurt et al., 2015). SIRT1 can also affect the functional state of osteoclasts and regulate bone turnover. Gurt et al. (2015) confirmed that SIRT1 inhibits osteoclast formation induced by NF-κB activator ligand (Tseng et al., 2011). SIRT1 can also inhibit osteoclast formation through deacetylation of FoxOs and reduce reactive oxygen species (ROS) levels, thereby improving oxidative stress-induced bone formation damage (Li Y. et al., 2014; Kim et al., 2015).

Furthermore, bone marrow stem cells can also differentiate into adipocytes. Sirtuin 1 can indirectly promote osteogenic differentiation by inhibiting adipogenic differentiation in the osteogenic induction of preosteoblasts and bone marrow stem cells (Zhou et al., 2016). In addition, SIRT1 is closely related to parathyroid hormone (PTH) and estrogen and indirectly regulates bone metabolism by interacting with hormones. Fei et al. (2015) found that SIRT1 inhibited the activation of metalloprotein 13 by PTH in SIRT1 gene knockout mice and osteoblasts, thus inhibiting osteogenic differentiation.

Interestingly, SIRT-1 can protect against age-related bone loss, whereas reducing its expression causes decreased bone formation in mice, further indicating that SIRT-1 is an important epigenetic regulator in aging bone cells (Herranz and Serrano, 2010; Cohen-Kfir et al., 2011). Carmeliet et al., showed that enhanced HIF-1α signaling increases Sirtuin 1-dependent deacetylation of the SOST promoter, resulting in decreased sclerostin expression and enhanced WNT/β-catenin signaling (Stegen et al., 2018). Increasing the activity of SIRT-1 protein in MSCs through the phytoestrogen resveratrol can lead to increased osteoblast differentiation and decreased adipocyte differentiation (Backesjo et al., 2009; Tseng et al., 2011; Sreng et al., 2019). In a mouse model of premature, resveratrol treatment improves trabecular bone structure and mineral density by enhancing the binding of SIRT-1 and laminin A (Liu et al., 2012). Overall, these studies show that SIRT-1 can reduce certain aging mechanisms in bone cells that contribute to bone aging.

### Histone Methylation

Histone methylation usually occurs at the lysine (K) and arginine (R) residues of histone N end, and unlike acetylation, methylation sites are characterized by transcription activation and inhibition; for example, methylation of histones H3 K4, K36, and K79 is related to transcription activation, and the methylation of H3K9, H3K27, and H4K20 is related to transcription inhibition (Xu et al., 2020). Histone methylation is regulated jointly by both methylases and demethylases; methylases include suppressor of variegation 3–9 (Drosophila) homolog 1 (SUV39H1), G9a and Enhancer of zeste homolog 2 (EZH2), while demethylases include Lysine-specific demethylase 1 (LSD1) and jumonji domain-containing protein (JMJD) (Separovich et al., 2020). Methyltransferases and demethylases regulate the expression of related genes in osteoblasts and osteoclasts (Table 3).

**TABLE 3:** Histone demethylases, target histones, and their roles in the osteoblast differentiation.

HDMS	Target histones	Target genes	Function
LSD1/KDM1A	H3K4me3	Wnt7B, BMP2	Inhibit osteoblast differentiation (Sun J. et al., 2018)
KDM2B	H3K4me3, H3K36me1/2	AP-2α	Involved in the proliferation and differentiation of early and late ameloblast cells as well as the differentiation of dentin (Fan et al., 2009)
KDM4A	H3K9me3	Sfrp4, C/EBPα	Promote adipogenic differentiation and inhibit osteoblastic differentiation of stem cells (Qi et al., 2020)
KDM4B	H3K9me3, H3K27me3	DLX	Promote osteoblast differentiation (Ye et al., 2012)
KDM5A	H3K4me3	BMP2, RUNX2	Inhibit osteoblast differentiation (Flowers et al., 2010)
JMJD3/KDM6B	H3K9me3, H3K27me3/2	HOX	Promote osteoblast differentiation (Ye et al., 2012; Hoang et al., 2016)
KDM7A	H3K9me2, H3K27me2	C/EBPα, Wnt pathway	Promote adipogenic differentiation and inhibit osteoblastic differentiation (Yang X. et al., 2019)
NO66	H3K4, H3K36	OSX	Inhibit osteoblast differentiation (Chen et al., 2015)
RBP2/JARID1A	H3K4me3/2	RUNX2	Inhibit osteoblast differentiation (Ge et al., 2011)
JMJD7	/	c-fos, Dc-stamp, CtsK, Acp5 and Nfatc1	Inhibit osteoclast differentiation (Liu Y. et al., 2018)

*LSD1, Lysine-specific demethylase 1; JMJD, jumonji domain-containing protein; WDR5, WD repeat-containing protein 5; KDM4B, lysine (k)-specific demethylases 4B; NFATc1, nuclear factor of activated T cell cytoplasmic 1.*

Enhancer of zeste homolog 2 is a methylransferase that trimethylates H3K27 and plays an inhibitory role in epigenetics (Dudakovic et al., 2018). Wei et al. (2011) reported that EZH2 inhibited the differentiation of MSCs into osteoblasts. Dudakovic et al. (2013) showed that EZH2 was downregulated in the process of osteoblastic differentiation. WD repeat-containing protein 5 (WDR5) is another methyltransferase, and that methylates H3K4 to accelerate osteoblast differentiation. ChIP experiment proved that WDR5 can be combined in the promoter regions of WNT1, RUNX2 and c-myc to regulate osteoblast differentiation through classical Wnt signaling pathways (Zhu et al., 2008).

Sun J. et al. found that LSD1, also known as KDM1A, is a key epigenetic regulator of osteoblast differentiation (Sun J. et al., 2018). *In vitro* mechanistic studies have shown that LSD1 deficiency increases the expression of BMP2 and WNT7B in osteoblasts and enhances bone formation, suggesting that LSD1 is a new regulator of osteoblast activity (Ye et al., 2012). JMJD3, a kind of H3K27 demethylase, is increasingly expressed in the process of osteoblast differentiation and regulates the bone-related genes Runx2, OSX, and OCN to promote osteoblast differentiation (Yang et al., 2013; Zhang F. et al., 2015). In osteoclasts, JMJD3 promotes the activation of the RANKL signaling pathway by prohibiting the methylation of H3K27 in the nuclear factor of activated T-cells (NFATC1) promoter region, thus promoting the differentiation of osteoclasts (Yasui et al., 2011).

## Non-Coding RNA

Non-coding RNA is a type of RNA that is transcribed from the genome but does not encode a protein (Yang et al., 2020). According to the length of RNA, it non-coding RNA is divided into three types: (1) a length less than 50 nt, including microRNAs (miRNAs), small interfering RNAs (siRNAs), and new non-coding small RNAs (priRNAs); (2) a length ranging from 50 to 500 nt, including ribosomal RNA (rRNA) and transfer RNA (tRNA); and (3) a length greater than 500 nt, including long non-coding RNAs (lncRNAs) and circular RNAs (circRNAs), which differ from traditional linear RNA (Li et al., 2020; Yang et al., 2020). In the past, scholars often ignored the role of non-coding RNA and regarded it as “junk RNA.” However, with the progress of scientific thinking and laboratory technology, an increasing number of studies have reported an association between the abnormal expression of non-coding RNA and the development of bone metabolic diseases (Letarouilly et al., 2019; Yang et al., 2020). If we determine key role of non-coding RNA in the process of bone metabolism, we will be able to design drugs for targeted therapy that are expected to fundamentally block and treat diseases associated with bone metabolism. In this paper, the molecular biological mechanism by which non-coding RNA regulates bone metabolism is reviewed to provide a reference for biological research and the clinical treatment of osteoporosis.

### lncRNA

Non-coding RNAs with a length of more than 500 nt are defined as lncRNAs. Initially, lncRNAs were not considered a transcriptional product of RNA (Liu et al., 2019). However, recent studies have identified roles for lncRNAs in many important biological processes, including genomic imprinting, chromosome modification, chromosome silencing, transcriptional interference and transcriptional activation (Delas and Hannon, 2017; Li et al., 2020). The abnormal expression of lncRNAs will induce uncontrolled transcription and abnormal expression of related proteins, eventually leading to the development of human disease (Kazemzadeh et al., 2015; Delas and Hannon, 2017).

**FIGURE 5:**
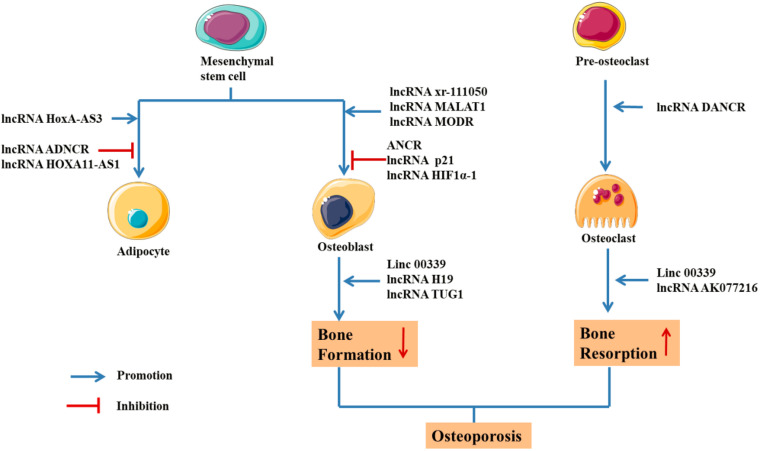
Schematic drawing of functional lncRNAs implicated in osteoporosis (Keniry et al., 2012; Zhu and Xu, 2013; Chan et al., 2014; Che et al., 2015; Tong et al., 2015; Xiao et al., 2015; Xu et al., 2015; Huang et al., 2016; Liang et al., 2016; Zhu et al., 2016; Chen et al., 2017; Wang et al., 2017; Wei et al., 2017; Weng et al., 2017; Xia et al., 2017; Zhang et al., 2017; Feng X. et al., 2018; Feng L. et al., 2018; Li H. et al., 2018; Sun X. et al., 2018; Liu et al., 2019; Yang et al., 2019a; Zhu E. et al., 2019; Zhu J. et al., 2019).

The primary, secondary, and tertiary structures of ncRNA interact with RNA, DNA and proteins to exert the biological activity. However, lncRNAs are different from miRNAs because they lack a universal mechanism of action and regulate gene expression and protein synthesis through various pathways (Delas and Hannon, 2017). In a study of postmenopausal women with osteoporosis, 51 lncRNAs were abnormally expressed, with some participating in the pathological process of OP by regulating mRNA expression or osteoclast differentiation (Fei et al., 2018). Based on these results, RNAs regulate the process of bone regeneration by modulating RNAs and transcription factors (Table 4 and Figure 5; Kunej et al., 2014; Zhu et al., 2015).

**TABLE 4:** lncRNAs and their roles in the osteoblast differentiation.

LncRNAs	Target genes	Function
H19	miR-675, miR-141, miR-22	Promote osteoblastic differentiation (Keniry et al., 2012; Liang et al., 2016)
	CTCF/H19/HDAC pathway	Promote adipogenic differentiation (Chan et al., 2014; Huang et al., 2016)
LncRNA p21	Wnt/β-actin pathway	Promote osteoblastic differentiation (Xia et al., 2017)
Bmcob	SBP2	Promote osteoblastic differentiation (Sun X. et al., 2018)
HIF1α-AS1	HOXD10, SIRT1	Inhibit (Xu et al., 2015; Zhu J. et al., 2019)
LncRNA TUG1	Wnt/β-actin pathway	Promote osteoblastic differentiation (Chen et al., 2017)
XR-111050	RUNX2	Promote osteoblastic differentiation (Zhang et al., 2017)
DNACR	P38 MAPK pathway	Inhibit osteoblast differentiation (Tong et al., 2015)
AK-096529, uc003ups, AK05611	Smurf1, RUNX2	Promote osteoblastic differentiation (Zhu J. et al., 2019)
HOTAIR	BMP/TGF-β pathway	Inhibit osteoblast differentiation (Wei et al., 2017)
lncRNA MALAT1	RANK/RANKL/OPG pathway	Promote osteoblastic differentiation (Che et al., 2015)
MODR	MiR-454/RUNX2	Promote osteoblastic differentiation (Weng et al., 2017)
AK141205	CXCL13	Promote osteoblastic differentiation (Xu et al., 2015)
MEG3	MiR-133a-3p	Inhibit osteoblast differentiation (Wang et al., 2017)
ANCR	EZH2, RUNX2	Inhibit osteoblast differentiation (Zhu and Xu, 2013)
BDNF-AS	RUNX2	Inhibit osteoblast differentiation (Feng X. et al., 2018)
Plnc1	PPAR-g2	Promote adipogenic differentiation (Zhu E. et al., 2019)
ADINR	C/EBPα	Promote adipogenic differentiation (Xiao et al., 2015)
HoxA-AS3	EZH2	Promote adipogenic differentiation and inhibit osteoblastic differentiation (Zhu et al., 2016)
ORLNC1	ORLNC1-miR-296-PTEN pathway	Promote adipogenic differentiation and inhibit osteoblastic differentiation (Yang et al., 2019a)
Bmncr	BMP2, TAZ, RUNX2, PPARG	Promote osteoblastic differentiation and inhibit adipogenic differentiation (Li C. J. et al., 2018)
lncRNA NEAT1	miR-29b-3p	Promote osteoblastic differentiation
lncRNA TCONS_00041960	RUNX2	Promote osteoblastic differentiation and inhibit adipogenic differentiation (Shang et al., 2018)
LncRNA BDNF-AS	miR-204-5p, miR-125a-3p	Inhibit osteoblast differentiation (Feng X. et al., 2018)
Linc-ROR	miR-138, miR-145	Promote osteoblastic differentiation (Feng L. et al., 2018)

*BMSCs, bone mesenchymal stem cells; TGF, transforming growth factor; HIF, hypoxia-inducible factor; HOXD10, homeobox D10; RUNX2, runt-related transcription factor 2; DANCR, differentiation antagonizing non-protein coding RNA; MAPK, mitogen-activated protein kinase; Smurf, Smad ubiquitination regulator; PPAR, peroxidase proliferator activator receptor; RANKL, nuclear factor-κB ligand; CXCL, CXC motif chemokine; SBP2, selenocysteine insertion sequence-binding protein 2; CTCF, CCCTC-binding factor; HDAC, histone deacetylase; EZH2, Enhancer of zeste homolog 2; TAZ, transcriptional co-activator with PDZ-binding motif; PPARG, peroxisome proliferator-activated receptors G; C/EBPα, CCAAT/enhancer binding protein alpha; ROR, receptor tyrosine kinase-like orphan receptor.*

#### H19

The H19 gene is relatively conserved throughout evolution and plays an important role in regulating biological functions. As a precursor of miR-675, H19 produces two mature microRNAs (miR-675-5p and miR-675-3p) after cleavage by Drosha and Dicer. During the osteogenic differentiation of human MSCs, the expression of H19 and miR-675 is upregulated (Zhang et al., 2018). The upregulation of miR-675 not only downregulates TGF-β1 but also inhibits the phosphorylation of Smad3, thus downregulating HDAC4/5, leading to a decrease in HDAC levels and promoting osteogenesis (Keniry et al., 2012). As shown in the study by Liang et al. (2016) H19, an endogenous competitive ceRNA of miR-141 and miR-22, directly binds to these two miRNAs and blocks their inhibitory effect on the Wnt/β-Catenin pathway, thus promoting osteogenic differentiation. Huang et al. (2016) reported that overexpression of H19 and miR-675 inhibits adipose differentiation. Importantly, miR-675 binds to the 3′-untranslated region (UTR) of HDAC4-6, downregulating their expression and thus inhibiting adipose differentiation, which requires HDAC4-6.

**FIGURE 6:**
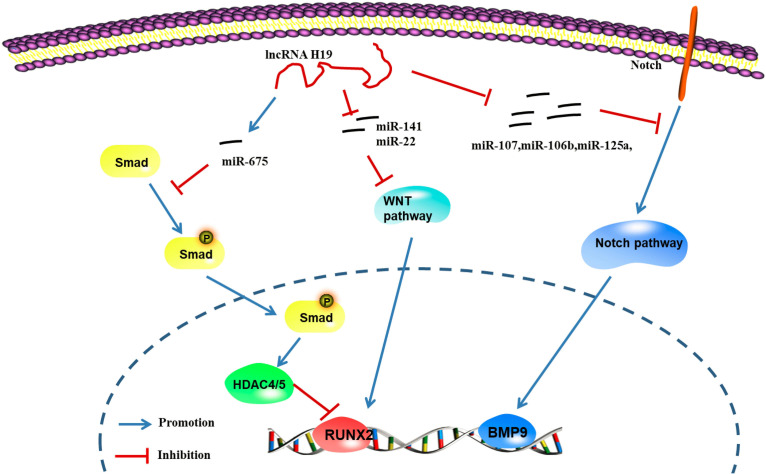
LncRNA H19 regulates the gene pathway of osteogenic differentiation through the lncRNA-miRNA-mRNA network (Keniry et al., 2012; Chan et al., 2014; Huang et al., 2016; Liang et al., 2016; Liao et al., 2020). H19 can up-regulate the expression of miR-675, further inhibit the phosphorylation of TGF-1 and Smad3, and downregulate the expression of Histone deacetylase 4/5 (HDAC4/5), and promote the expression of genes related to osteogenic differentiation; H19 can inhibit the expression of miRNAs (miR-141 and miR-22), promote Wnt/β-catenin signal transduction pathway, and promote osteogenic differentiation; H19 can regulate the expression of ligands such as Dll1, Dll3, Dll4, Jag1, and Jag2 in Notch signaling pathway by regulating the expression of miRNA (miR-107, miR-27b, miR-106b, miR-125a, and miR-17) to further promote the induction of the osteogenic differentiation by BMP9 (Zhang et al., 2018).

According to Chan et al. (2014) abnormal expression of the lncRNA H19 upregulates the expression of genes in the hedgehog signaling pathway and yes-associated protein 1 (YAP1), leading to abnormal osteoblast proliferation. Liu et al., identified a regulatory effect of the lncRNA H19 on the expression of delta-like ligand 1 (DLL1), delta-like ligand 3 (DLL3), delta-like ligand 4 (DLL4), Jagged 1 (JAG1) and Jagged 2 (JAG2) in the Notch signaling pathway by regulating the expression of downstream miRNAs (miR-107, miR-27b, miR-106b, miR-125a, and miR-17), thus promoting the expression of bone morphogenetic protein 9 (BMP-9) and inducing the osteogenic differentiation of MSCs (Liao et al., 2020). These studies confirmed that lncRNA H19 promotes osteogenic differentiation through the lncRNA/miRNA/mRNA network (Figure 6; Zhang et al., 2018).

#### lncRNA DANCR

Tong et al. (2015) reported the significant upregulation of the expression of lncRNA DANCR in blood mononuclear cells from patients with reduced BMD based on a qRTPCR analysis, and DANCR increased the expression of the IL6 and TNF-α mRNAs and proteins. Furthermore, DANCR induces the expression of IL6 and TNF-α in mononuclear cells to promote the bone resorptive activity of osteoclasts. The siRNA-mediated inhibition of DANCR reduces IL6 and TNF-α levels in blood mononuclear cells from postmenopausal women with a reduced bone density (Tong et al., 2015). Thus, DANCR is related to IL6 and TNF-α levels in blood mononuclear cells from patients with a reduced bone density. From the perspective of immunity, OP is considered a chronic immune-mediated inflammatory disease, in which the production of cytokines and activation of the inflammatory response trigger the immune system, resulting in increased osteoclast activity and disordered bone transformation to increase bone absorption and produce OP.

#### ANCR

Anti-differentiation non-coding RNA (ANCR) is a new type of long chain non-coding RNA. Its expression is downregulated during stem cell differentiation, which is necessary to maintain osteoblasts in an undifferentiated state. ANCR is closely related to osteoblast differentiation (Zhu and Xu, 2013). Recently, siRNA-mediated silencing of ANCR was shown to increase the levels of osteoblast differentiation markers, such as alkaline phosphatase and osteocalcin, while overexpression of ANCR reduced the expression of these markers. Regarding the mechanism, previous studies have confirmed that ANCR regulates RUNX2 expression by recruiting EZH2. EZH2 mainly catalyses H3-lysine-27 trimethylation at the RUNX2 gene promoter to inhibit RUNX2 expression and subsequent osteoblast differentiation. Further studies also confirmed the direct relationship between ANCR and EZH2.

#### MALAT1

Xiao et al. (2017) confirmed that metastasis-associated lung adenocarcinoma transcript 1 (MALAT1) promotes the osteogenic differentiation of aortic valve stromal cells in individuals with calcified aortic valve disease (CAVD). Furthermore, MALAT1 functions as a sponge for miR-204, leading to the upregulation of Smad4 expression, which promotes the expression of alkaline phosphatase and the downstream molecule osteocalcin to induce bone formation and mineralization. As shown in the study by Dong et al. (2015) using bone tumor cells, knockout of MALAT1 inhibited the expression of proliferating cell nuclear antigen (PCNA), matrix metalloproteinase-9 (MMP-9), P85α, and Akt, thus reducing the proliferation of osteoblasts. Che et al. (2015) studied the regulation of RANK/RANKL/OPG signaling in the human osteoblast cell line hFOB 1.19 and found that the lncRNA MALAT1 regulated the RANK/RANKL/OPG pathway in the pathological state of an imbalanced bone metabolism, thus activating and remodeling osteoclast activity in the bone model. Zheng et al., also successfully established an osteoporosis model in SD rats and detected the expression of MALAT1 in rats with osteoporosis and normal rats using real-time polymerase chain reaction. The lncRNA MALAT1 was expressed at low levels in rats with osteoporosis (Zheng et al., 2019). Moreover, lncRNA MALAT1 inhibits the osteoblastic differentiation of BMSCs by increasing the activation of the MAPK signaling pathway, thus promoting the process of osteoporosis (Zheng et al., 2019).

#### lncRNA p21

Bone marrow mesenchymal stem cells (BMMSCs) are pluripotent stem cells with the ability to differentiate into osteoblasts. The downregulation of lncRNA p21 stimulates BMMSCs to secrete the vascular endothelial growth factor, basic fibroblast growth factor, and insulin-like growth factor and induces the expression of β-Catenin protein, thus promoting the osteoblast differentiation of BMMSCs (Xia et al., 2017).

#### lncRNA TUG1

Chen et al. (2017) showed that lncRNA TUG1 can promote the expression of β-Catenin, MMP3 and Caspase-3, inhibit the expression of BCL-2 and proteoglycan, inhibit the apoptosis and aging of osteoblasts and promote cell proliferation through the Wnt/β-Catenin signaling pathway (Chen et al., 2017).

#### lncRNA HOTAIR

Bone morphogenetic proteins are a member of the TGF-β superfamily and have many subtypes. The BMP TGF-β signaling pathway can regulate the expression of the RUNX2 gene in BMMSCs through the classical Smad pathway and non-classical p38 pathway, thus regulating the differentiation and function of osteoblasts and playing an important role in the bone metabolism balance in osteoporosis. Wei et al. (2017) showed that lncRNA HOTAIR regulates miR-17-5p and Smad7 through the BMP/TGF-β signaling pathway, as well as osteogenic differentiation and proliferation.

#### lncRNA HIF1α-AS1

As shown in the study by Xu et al. (2015) lncRNA HIF1α-AS1 activates the BMP/TGF-β pathway and interferes with SIRT1 expression. Furthermore, lncRNA HIF1α-AS1 downregulates HOXD10 and interferes with histone acetylation, leading to the inhibition of osteoblast differentiation (Xu et al., 2015). Based on these results, HIF1α-AS1 is the key factor in osteoblast differentiation and is expected to become a gene therapy target for osteoporosis.

#### lncRNA xr-111050

Zhang et al. (2017) studied the expression profile and function of lncRNAs during the differentiation of BMMSCs into osteoblasts or osteoclasts and found that lncRNA xr-111050 regulates this differentiation process by regulating the MAPK signaling pathway.

#### Other Long Non-coding RNAs

The pathogenesis of various metabolic bone diseases represented by OP, rheumatoid arthritis-related bone loss, Paget’s bone disease, diabetic osteoporosis and other diseases may be related to osteoclast hyperactivity (Galson and Roodman, 2014; Jiao et al., 2015). Notably, lncRNAs enhance bone resorption by promoting osteoclast formation. The expression of lncRNA AK077216 was significantly upregulated during osteoclastogenesis. Additionally, the upregulation of lncRNA AK077216 expression can increase osteoclast formation, promote osteoclast function and increase bone resorption by regulating the expression of NFATc1. Moreover, lncRNAs can increase osteoclast activity. In addition to regulating osteoclast activity, lncRNAs can also promote osteoclast formation. Peripheral blood mononuclear cells are precursors of osteoclasts, and they directly participate in the formation of osteoclasts and secrete Osteoclast-6 and TNF-α (Cohen-Solal et al., 1993; Fujikawa et al., 1996). In conclusion, lncRNAs can promote the production and proliferation of osteoclasts, increase the activity of osteoclasts and promote the development of OP by regulating the expression of proteins in the Notch signaling pathway, transcription factors and immune factors (Liu et al., 2019).

Researchers gradually recognized that lncRNAs play key roles in various biological processes, including cell growth, transcriptional regulation and differentiation. Maladjusted lncRNAs are closely related to human diseases, including bone and muscle diseases and cancer. Notably, lncRNAs play important roles in the pathogenesis and treatment of OP (Figure 5). The mechanism by which lncRNAs regulate bone metabolism through different signaling pathways is still being investigated. With the development of research technology and methods, the key regulatory mechanisms underlying the effect of lncRNAs on the bone metabolism signaling pathways will be further clarified, and these results would have important potential clinical applications in the treatment of OP.

### MircoRNAs

**FIGURE 7:**
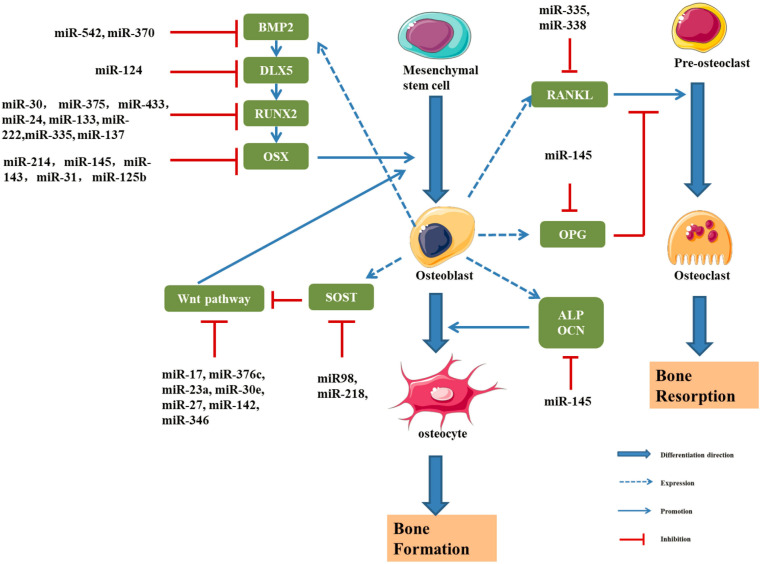
Schematic drawing of miRNAs implicated in osteoblast differentiation (Li et al., 2009, 2012, 2015; Hu et al., 2011; Wu et al., 2012; Zhang et al., 2012; Grunhagen and Ott, 2013; Liu et al., 2013, 2020; Wang et al., 2013; Kato et al., 2014; Kureel et al., 2014; Li E. et al., 2014; Qi and Zhang, 2014; Sun et al., 2014, 2016; Yang et al., 2014, 2017; Xiao et al., 2016; Zheng et al., 2017; Xie and Cao, 2019; Zhang L. et al., 2019; Krzeszinski et al., 2020; Yan et al., 2020).

MicroRNAs (miRNAs) are a type of endogenous non-coding small, single-stranded RNA of approximately 22 nucleotides in length. It is complementary to the site of the 3′ untranslated region of the target gene mRNA and binds through sequence-specific base pairing (Gernapudi et al., 2016; Ghayor and Weber, 2016; Letarouilly et al., 2019). Notably, miRNAs regulate the bone metabolism by regulating the target genes related with osteogenic differentiation (Table 5 and Figure 7; Dou et al., 2016; Letarouilly et al., 2019).

**TABLE 5:** miRNAs and their roles in the osteoblast differentiation.

miRNAs	Target genes	Function
miR-146a	NF-κB pathway	Promote osteoblastic differentiation (Zheng et al., 2017)
miR-214	OSX, WNT pathway	Inhibit osteoblastic differentiation (Grunhagen and Ott, 2013)
miR-4448, miR-4708, miR-4773	SMAD1 and SMAD4	Inhibit osteoblastic differentiation (Kato et al., 2014)
miR-30	SMAD1 and RUNX2	Inhibit osteoblastic differentiation (Wu et al., 2012)
miR-34a	TGIF	Inhibit osteoclast growth (Krzeszinski et al., 2020)
miR-542-3p	BMP7	Inhibit osteogenic differentiation and promote apoptosis of osteoblasts (Kureel et al., 2014)
miR-346	GSK3β, c-Myc	Promote osteoblastic differentiation (Wang et al., 2013)
miR-26a	HMGA1	Promote osteoblastic differentiation and inhibit adipogenic differentiation (Li et al., 2012; Yan et al., 2020)
miR-548d-5p	PPARγ	Promote osteoblastic differentiation and inhibit adipogenic differentiation (Sun et al., 2014)
miR-99a	KDM6B, HOXC6-1, HOXA10, HOXB2 and HOXC10	Promote osteoblastic differentiation (Xie and Cao, 2019; Zhang L. et al., 2019)
miR-21	Spry	Promote osteoblastic differentiation (Yang et al., 2017)
miR-145	OSX	Inhibit osteoblastic differentiation (Sun et al., 2016)
miR-2861	HDAC5	Promote osteoblastic differentiation (Li et al., 2009; Hu et al., 2011)
miR-3960	HOXA2	Promote osteoblastic differentiation (Hu et al., 2011)
miR-433	RUNX2	Inhibit osteoblastic differentiation (Kim et al., 2013)
miR-335	RUNX2	Inhibit osteoblastic differentiation (Zheng et al., 2017)
miR-106b-5p and miR-17-5p	Smad5	Inhibit osteoblastic differentiation (Fang et al., 2016)
miR-335-5p	DKK1	Promote osteoblastic differentiation (Zhang J. et al., 2011)
miR-29a	DKK1, Krm2, and sFRP2	Promote osteoblastic differentiation (Kapinas et al., 2010)
miR-218	SOST, DKK2, and sFRP2	Promote osteoblastic differentiation (Hassan et al., 2012)
miR-143, miR-31	OSX	Inhibit osteoblastic differentiation (Zhang et al., 2012; Li E. et al., 2014; Liu et al., 2020)
miR-101, miR-132	PI3K/AKT/mTOR pathway	Promote osteoblastic differentiation (Qi and Zhang, 2014)
miR-17	TCF/Wnt pathway	Inhibit osteoblastic differentiation (Liu et al., 2013)
miR-216	PI3K/AKT pathway	Promote osteoblastic differentiation (Xiao et al., 2016)
miR-194	STAT1	Promote osteoblastic differentiation (Li et al., 2015)
miR-96	EGFR signaling	Promote osteoblastic differentiation (Yang et al., 2014)
miR-23	MARK pathway	Inhibit osteoblastic differentiation (Jiang et al., 2020)
miR-375	RUNX2	Inhibit osteoblastic differentiation (Du et al., 2015)
miR-153	BMPRII	Inhibit osteoblastic differentiation (Cao et al., 2015)
miR-124	DLX	Inhibit osteoblastic differentiation (Zhang C. et al., 2015)
MiR-125b	OSX	Inhibit osteoblastic differentiation (Chen et al., 2014)

*mTOR, mammalian target of rapamycin; SMAD1, SMAD Family Member 1; TGIF, transforming growth factor-β induced factor; BMP7, Bone morphogenetic protein 7; GSK3β, Glycogen synthase kinase-3β; c-Myc, MYC proto-oncogene; HMGA1, high mobility group A; PPARγ, peroxisome proliferator activated receptor γ; Spry, Sprouty; OSX, osterix; HDAC 5, histone deacetylase 5; HOXA2, Homeobox A 2; RUNX2, Runt-related transcription factor 2; DKK1, Dickkopf 1; DKK1, Dickkopf 2; Krm2, Kremen2; sFRP2, secreted frizzled-related protein 2; SOST, sclerostin; STAT1, signal transducer and activator of transcription 1; BMPRII, bone morphogenetic protein receptor II; DLX, distal-less homeobox gene.*

#### miR-145

Sun et al. (2016) found that a decrease in the miR-145 level induces the expression of RUNX2, OSX, and β-Catenin, thus promoting osteogenic differentiation. Overexpression of miR-145 inhibits osteogenic differentiation by negatively regulating OSX expression. A clinical study reported lower expression of miR-145 in patients with osteodysplasia than in healthy controls (Wang et al., 2012). Dynamic detection of miR-145 levels during osteogenic differentiation showed that miR-145 was negatively correlated with the expression of forkhead box protein O1 (FOXO1), and the dual luciferase assay showed that miR-145 directly and negatively regulated FOXO1 expression (Wang et al., 2012).

#### miR-3960 and miR-2861

Li et al. (2009) showed that the expression of miR-2861 was increased in ST2 cells and overexpression of miR-2861 could enhance the differentiation ability of BMP-2. Studies also found that miR-3960 at the same locus of miR-2861 could inhibit the expression of HOXA2, an inhibitor of RUNX2, so as to promote the osteogenic differentiation. Moreover, results show that RUNX2 can bind to the promoters of miR-3960/miR-2861 to promote the expression of miR-3960/miR-2861. Therefore, RUNX2 and miR-3960/miR-2861 constitute a positive feedback cycle to continuously enhances the osteogenic differentiation ability of ST2 cells (Hu et al., 2011).

#### miR-21

The Spry family is composed of Spry1, Spry2, Spry3, and Spry4, which are highly conserved between humans and rats. In the osteogenic differentiation of BMSCs, the expression of Spry1 was downregulated and the expression of RUNX2 and OSX was upregulated. Yang et al. (2017) studied the functional axis of miR-21/Spry1 in human BMSCs and found that overexpression of miR-21 promoted osteogenic differentiation by inhibiting the expression of Spry1. The overexpression of miR-21 significantly increased alkaline phosphatase activity significantly. Moreover, miR-21 can inhibit osteogenic differentiation by downregulating Spry1 and upregulating RUNX2 and OSX to further modulate the inhibitory effect of Spry1 on osteogenic differentiation (Yang et al., 2017).

#### Involvement of miRNA During Bone Aging

It has been found that a variety of miRNAs are involved in the aging process of bone tissue (Liu M. et al., 2018; Cakouros and Gronthos, 2020). The regulation of miRNA on age-related bone tissue provides new theoretical basis for the clinical treatment of bone density reduction and age-related osteoporosis caused by bone aging.

Ruben et al., found that the expression of miR-219a-5p in bone tissues of aged mice decreased, and it involved in the aging process by regulating the expression of the target gene retinoic acid receptor-related orphan receptor beta (Rorβ) (Aquino-Martinez et al., 2019). Liu et al. found that serum miR-96 was significantly up-regulated in elderly patients with osteoporosis. Further studies found that after overexpression of miR-96, the thickness and number of trabecular bone in young mice were significantly reduced (Liu H. et al., 2018). Xu R. et al. found that the level of miR-31a-5p in the exosomes of BMSCs in aged rats was significantly increased. Inhibiting the expression of miR-31a-5p can effectively reduce bone loss and reduce osteoclast activity in aged rats (Xu R. et al., 2018). Similar studies comparing young and old human BMSC, uncovered miR 199b-5p was deregulated during BMSC aging, which is predicted to target SIRT1 (Peffers et al., 2016).

With the aging of bone tissue, adipose tissue in the bone marrow accumulates and the number of mesenchymal stem cells in the intercellular phase increases. Fan et al. (2018) found that the overexpression of miR-1292 accelerates the senescence of human adipose derived stem cells (hADSCs) and inhibits bone formation through Wnt/β-catenin signaling pathway. Li H. et al. found that miR-10b inhibits the adipose differentiation of hADSCs through the TGF-β pathway (Li H. et al., 2018). The aging-related gene Smurf1 in BMSCs of aged mice is significantly increased. miR-17 inhibits the expression of Smurf1 through the p53/miR-17/Smurf1 signaling pathway, and promotes the osteogenic differentiation of aging BMSCs (Liu et al., 2015).

#### Other miRNAs

MiR-143 can downregulate the expression of OSX and inhibit the osteogenic differentiation of MC3T3-E1 cells (Li E. et al., 2014). Some studies have found that miR-125b could affect the proliferation and osteogenic differentiation of BMMSCs by regulating the expression of OSX (Chen et al., 2014). In addition, miR-138, miR-31, miR-142, miR-148, and miR-637 could inhibit the expression of OSX (Zhang et al., 2012; Liu et al., 2020). Chen et al. (2016) found that serum miR-30b-5p level was significantly down-regulated in postmenopausal women, and the results were consistent in the corresponding rat model. BMSCs from ovariectomized mice and sham operated mice were compared, and miR-26a expression was significantly decreased in the ovariectomized group. This experiment confirmed the ability of miR-26a to induce the osteogenic differentiation of BMSCs in mice and confirmed that miR-26a was a negative regulator of osteoporosis (Yan et al., 2020).

Further study of miRNAs associated with osteogenic differentiation will help us better understand the pathogenesis of bone metabolism and OP. However, at present, the study of miRNA in the pathological mechanism of osteoporosis is still very limited. The functions of numerous unknown miRNAs require further exploration by researchers. For known miRNAs, the regulatory mechanism, selection of downstream target mRNAs and association with related diseases also requires further study.

### circRNA

Compared with other ncRNAs, circRNAs replace the traditional structure pattern of the 5′-end cap and 3′-end polyadenylate tail with the special structure of a continuous covalent closed loop, and they have higher conservation and stability (Chen and Yang, 2015; Yang and Duan, 2016). According to numerous studies, circRNAs participate in the occurrence and development of many diseases by regulating gene transcription, translation, splicing and other key steps (Rong et al., 2017; Xu S. et al., 2018). Accumulating research on circRNAs has revealed their important roles in diseases related to bone metabolism (Table 6; Zhu et al., 2020).

**TABLE 6:** Circular RNAs (circRNAs) and their roles in the osteoblast differentiation.

circRNAs	Targets	Functions
circVANGL1	miR-2l7	Promote osteoblastic differentiation (Yang et al., 2019b)
circ_003795	miR-504-3p	Promote BMSCs proliferation (Ren et al., 2019)
circ_0005105	miR-26a	Promote osteoblastic differentiation and inhibit adipogenic differentiation (Wu et al., 2017)
circ_0045714	miR-193b	Promote chondrocyte proliferation (Li B. F. et al., 2017)
circRNA533l	miR-204	Inhibit osteoblastic differentiation
circRNA CDRlas	miR-7	Inhibit osteoblastic differentiation
circRNA NFATCl	miR-4483	Promote osteoblastic differentiation
circRNA IGSFll	miR-199b-5p	Inhibit osteoblastic differentiation (Zhang M. et al., 2019)
circRNA RUNX2	miR-203	Promote osteoblastic differentiation (Yin et al., 2018)
circ_0127781	miR-210, miR-335	Inhibit osteoblastic differentiation (Mizuno et al., 2009; Zhang J. et al., 2011)
circ_0074834	miRNA-942-5p	Promote osteoblastic differentiation (Ouyang et al., 2019)
circ_33287	miR-214-3p	Promote osteoblastic differentiation (Peng et al., 2019)
CDR1as	miR-7-5p/Wnt 5B	Promote adipogenic differentiation and inhibit osteoblastic differentiation (Chen G. et al., 2020)
CircUSP45	miR-127-5p	Inhibit BMSCs proliferation (Kuang et al., 2019)

Dou et al. (2016) identified the differential expression of 518 lncRNAs, 207 mRNAs, 24 circRNAs, and 37 miRNAs at each stage of osteoclast differentiation. Qian et al. (2017) confirmed that BMP-2 induces osteogenic differentiation through circ191422/circ5846. Li X. et al. found that estrogen receptor (ER) β deficiency can inhibit the osteogenic differentiation of BMMSCs (Li X. et al., 2017). Silencing ERβ can cause the differential expression of some circRNAs in BMSCs and downregulate the expression of osteogenic related proteins at mRNA and protein levels. The RNA-SEQ analysis revealed the differential expression of 146 circRNAs, including 68 downregulated and 78 upregulated circRNAs. Subsequently, circRNAs were shown to play an important role in the osteogenic differentiation of BMMSCs. Zhang M. et al. found that during the osteogenic differentiation of BMMSCs, a total of 3938 circRNAs were upregulated and 150 circRNAs were downregulated compared with undifferentiated cells. Furthermore, the parental genes of differentially expressed circRNAs were associated with osteogenesis, suggesting that these circRNAs may play a role in the osteogenic differentiation of BMMSCs (Zhang M. et al., 2019). The silencing of circRNA IGSF11 promoted the differentiation of osteoblasts and increased the expression level of miR-199b-5p, indicating that the interaction of this circRNA and miRNA exerted a positive effect on the osteogenic differentiation of human BMMSCs. In addition, hsa_circ_0127781 interacts with miR-210-5p and miR-335-5p; miR-210 positively regulates osteogenic differentiation by inhibiting Activin A receptor 1B (AcvR1b) and miR-335 promotes osteogenic differentiation by activating the Wnt signaling pathway by downregulating DKK-1 (Mizuno et al., 2009; Zhang J. et al., 2011). Therefore, hsa_circ_0127781 may inhibit the osteogenic differentiation of BMSCs by functioning as an miRNA “sponge.”

Yin et al. (2018) investigated the prevention and treatment of osteoporosis and found that circRUNX2 interacts with miR-203, increases the expression of RUNX2, and inhibits osteogenic differentiation during the osteogenic differentiation of human BMMSCs. Yang et al. found that circVANGL1 regulates RUNX2 expression by absorbing miR-2l7 and accelerates osteogenic differentiation (Yang et al., 2019b). Ren et al. (2019) stimulated BMMSCs with calcitonin gene-related peptide (CGRP) and identified 58 differentially expressed circRNAs, of which 44 were downregulated and 14 were upregulated; among these differentially expressed circRNAs, mmu-circRNA 003795 expression was significantly increased and mmu-miR-504-3p expression was increased. Based on these results, circRNAs play an important role in the CGRP-induced proliferation of BMMSCs and highlight the regulatory mechanism of circRNAs, which provides a new direction for studying the osteogenic differentiation and proliferation of BMMSCs.

In conclusion, the biological functions of circRNAs in bone metabolism-related diseases have not yet been elucidated. Therefore, more comprehensive and in-depth studies of circRNAs are required to provide effective new methods, new approaches and new ideas for the diagnosis, treatment and prognosis of bone metabolism-related diseases.

## Summary

Bone not only supports the body and protects the internal organs but also has a variety of metabolic functions, particularly in maintaining the mineral balance of the body. Bone tissue is always in a state of dynamic balance between bone resorption and bone formation called bone remodeling. When bone resorption exceeds bone formation, bone loss will occur, leading to osteoporosis in severe cases. Epigenetic mechanisms refer to all heritable regulatory pathways that affect gene expression without altering the DNA sequence, including DNA methylation, histone modification, chromatin remodeling and ncRNAs, which play important roles in many diseases, including osteoporosis. An in-depth study of these epigenetic mechanisms will provide a better understanding of the pathogenesis of abnormal bone metabolism and osteoporosis. However, the understanding of the epigenetics of bone remodeling abnormalities is currently very limited. A large number of unknown functions must be discovered and further explored by scholars. Additionally, known epigenetic regulatory factors, epigenetic regulatory mechanisms and the relationship between their downstream target genes and related diseases require further study, and the mechanisms of DNA modification and methylation remain to be elucidated. Nevertheless, the identification of specific biomarkers related to osteoporosis will substantially improve the clinical diagnosis and treatment of the disease. The wide application of epigenetic microarrays, high-throughput sequencing and other new technologies will help establish a complete epigenetic spectrum of normal bone and bone diseases based on the whole genome. Genetic markers of disease prevention will help identify clinical phenotypes. Additional research in this area will further reveal the biological bases of the basic mechanisms of bone remodeling and the delicate balance between anabolism and catabolism in bone tissue, providing new targets for the diagnosis and treatment of common bone remodeling disorders.

## Data Availability Statement

All data generated or analyzed during this study are included in this article. All data included in this study are available upon request by contact with the corresponding author.

## Author Contributions

FX and WL conceived and designed the study. FX, WL, XY, and LN performed the data collection and analysis. FX, LC, and GL interpreted the data and wrote the manuscript. Specially, XY and GL made great contributions in the process of revising this manuscript. All authors read and approved the final manuscript.

## Conflict of Interest

The authors declare that the research was conducted in the absence of any commercial or financial relationships that could be construed as a potential conflict of interest.
